# Early prevention of trauma-related infection/sepsis

**DOI:** 10.1186/s40779-016-0104-3

**Published:** 2016-11-08

**Authors:** Xiao-Yuan Ma, Li-Xing Tian, Hua-Ping Liang

**Affiliations:** State Key Laboratory of Trauma, Burns and Combined Injury, Research Institute of Surgery, Daping Hospital, Third Military Medical University, Chongqing, 400042 China

**Keywords:** Prevention, Trauma, Infection, Sepsis

## Abstract

Trauma still represents one of the major causes of death worldwide. Despite the reduction of post-traumatic sepsis over the past two decades, the mortality of septic trauma inpatients is still high (19.5–23 %). Early prevention of sepsis development can aid in the subsequent treatment of patients and help improve their outcomes. To date, the prevention of trauma-related infection/sepsis has mainly included infection prevention (e.g., surgical management, prophylactic antibiotics, tetanus vaccination, immunomodulatory interventions) and organ dysfunction prevention (e.g., pharmaceuticals, temporary intravascular shunts, lung-protective strategies, enteral immunonutrition, acupuncture). Overall, more efficient ways should be developed to prevent trauma-related infection/sepsis.

## Background

The mortality rate of trauma is still very high and is increasing, according to the World Health Organization. It is predicted that post-traumatic mortality will be a major cause of death in 2020. Traffic injuries commonly seen in civilian trauma patients are the leading cause of pre-hospital death [1, 2]. Combat-related injuries, which are a special form of trauma, will also have higher mortality rates if the wounded do not receive timely battlefield surgery and subsequent treatments [3]. Both pro- and anti-inflammatory responses are involved in the post-traumatic pathologic process, and they increase the risk of acute respiratory distress syndrome (ARDS), sepsis, and multiple organ failure (MOF). Early prevention of the development of sepsis following trauma can reduce the risk of both sepsis and multiple organ dysfunction syndrome (MODS) and can improve the patients’ outcomes.

The greatest danger after hemorrhage in both civilian and combat-related injuries is sepsis. Sepsis 3.0 was put forward by Professor Craig Coopersmith, chairman of the Society of Critical Care Medicine, at the Chinese Medical Association (CMA) ninth intensive medical conference in 2015. The experts suggested that the new definition of sepsis should focus more attention on organ dysfunction (OD). Thus, Sepsis 3.0 is composed of two parts: (1) Infection and (2) Sequential Organ Failure Assessment (SOFA) ≥ 2. As long as the above two conditions coexist, regardless of which occurs first, then sepsis will be diagnosed. According to the new definition of sepsis, the prevention of trauma-related infection/sepsis correspondingly includes the prevention of both infection (wound infection, primarily nosocomial infection) and OD.

### Infection prevention

Preventing infection following trauma basically involves preventing wound and nosocomial infection. Wound care methods commonly include surgical management (e.g., disinfection, debridement, profuse irrigation and wound cleansing, negative-pressure wound therapy, wound drainage, appropriate wound closure) and the administration of pharmaceuticals (e.g., prophylactic antibiotics, tetanus vaccination, immunomodulatory interventions). The prevention of nosocomial infection is another aspect of infection prevention. Immune dysregulation is a well-described consequence of trauma and can increase the risk of nosocomial infection. Regional proper clinical protocols and hygiene are the correct methods according to the accepted prevention principles and include the following measures: chlorhexidine, hydrocortisone, detrusor botulinum toxin A injection, enteral nutrition and management of tube system, which are used to prevent ventilator associated pneumonia (VAP), central line-associated bloodstream infection and urinary tract infection (UTI).

### Surgical management

#### Hair removal and skin disinfection

Hair is an autologous source of wound contamination, and removing hair from a wound can avoid its entanglement during suture and closure [4]. The type and time of shaving have been shown to be necessary in reducing the chance of infection. The infection rate of surgical wounds after preparing the skin with electric clippers is markedly lower than it is after preparing the skin with a razor [5]. Moreover, shaving the hair before wound repair is associated with a higher risk of surgical site infection than is clipping the hair immediately [6]. Although the use of antiseptic agents containing iodophor or chlorhexidine can suppress a broad spectrum of organisms and bacterial proliferation, they may damage wound defenses and promote the development of infection [7]. Consequently, the reasonable application of antiseptic agents to the wound should be considered.

#### Debridement

Wound debridement is the most common surgery used in both conflict and civilian cases. The first surgical treatment in war surgery at the first echelon hospital is debridement without primary closure [3]. The US military recommends repeat debridement and irrigation every 24–48 h before wound cleaning [8]. Debridement can remove devitalized and severely contaminated tissues and can prevent infection, and the basic principles of wound debridement are well accepted in the field of surgical management [9, 10]. However, Edlich et al. [4] suggested that less tissue debridement had been associated with a lower rate of wound infection. Thus, it is important to identify the definite limits of dead tissue, similar to the “4C” guidelines (color, consistency, contraction, circulation) of muscle viability. In the case of complex traumatic hand injuries, meticulous initial debridement of nonviable tissue and skeletal stabilization are paramount in preventing hand infection [11]. Multiple debridements will be necessary if significant contamination is present.

#### Mechanical cleansing

Early and thorough irrigation following wound debridement is one of the important steps in the basic principles of the management of war wounds [3, 8]. Gentle irrigation with low pressure and normal saline will wash out any residual debris and clot and dilute any bacterial load, whereas high-pressure irrigation (7 psi, pounds per square inch) is applied to dirty or heavily contaminated wounds [3, 12]. Additionally, mechanical cleaning with high-pressure irrigation may effectively decrease the level of bacterial contamination and reduce the incidence of wound infection [4, 13].

#### Negative-pressure wound therapy (NPWT)

NPWT systems (also referred to as vacuum-assisted wound closure) are composed of an open-pore sponge, semi-occlusive dressing, and negative pressure source and are commonly available in the US [10, 14]. Negative pressure ranging from −50 to −200 mmHg may be effective in higher-risk infective wounds [15]. NPWT has the frequently cited advantage of bacteria clearance from the wound environment. It was shown to reduce the bacterial bio-burden in wounds in the animal open fracture model contaminated with gram-negative bacilli. The colonization of gram-positive cocci (e.g., *Staphylococcus aureus*) also exists [16]. In addition, NPWT has more benefits than dressings do in the setting of wound infection [15–17]. Patients with persistent drainage treated with NPWT for at least 5 days had a lower rate of wound infection and a shorter period of drainage than did patients in the compressive dressing group [15]. Several studies have shown that the wound infection rate in the patients who adopted NPWT was significantly lower than that in patients in the wet-to-dry (WTD) group [16, 17]. In military fields, using NPWT during intercontinental aeromedical evacuation of combat casualties may also provide many benefits, such as earlier wound closure, lower infection rates, and better pain management [8, 18].

#### Wound drainage

Thorough wound drainage following debridement and irrigation is one of the steps in the basic principles of the management of combat-related injuries [3]. Traditional drains are commonly used within 24 h in wounds with deep cavities and dead space. Stannard et al. [15] evaluated the efficiency of NPWT for the management of persistent wound drainage. In addition, Rispoli et al. [19] reported a new technique, combining NPWT with traditional drainage, that allowed the conversion of deep cavitary defects to superficial defects to facilitate drainage. Further, deep wound infection was better controlled, and there were no complications detected such as abscess formation, tube-associated skin necrosis, or sepsis.

#### Wound dressings

A bulky absorbent dressing or cotton wool is necessary for an adequately excised wound. Bandaging wounds with sterile dressings is commonly used in initial care on the battlefield. Silver nitrate solution is routinely applied to dressings following burns [3, 8]. WTD dressings were suggested to be the standard of method for soft-tissue defects and open wounds in the past. Because WTD is associated with increased patient pain, healthcare costs and risk of nosocomial infections, safe and effective wound dressings are required [10]. Guthrie et al. [20] compared 3 dressings, Inadine® (USA), Acticoat® (Hull, UK) and Activon Tulle (Nottingham, UK) in a rabbit model of contaminated forelimb muscle injury. They found that the Inadine and Acticoat groups had significantly lower bacterial counts.

#### Wound closure

It is important that the wound be closed as soon as doing so is safe, but not before and not long thereafter [3, 21]. Traumatic laceration wounds (≤5 cm) without signs of infection can be closed immediately, and disinfected wounds may be closed up to 24 h afterwards (based on the Friedrich dogma); wounds with active signs of infection should undergo secondary closure after 3–5 days [22]. There is no powerful evidence to demonstrate that traumatic wounds should not be sutured after 6 hours. Baar et al. [23] conducted a prospective cohort study and showed that the duration of the wound (older or younger than 6 hours) was not a critical factor in the decision of wound closure. Contaminated wounds should never be primarily closed. Delayed primary closure (DPC) appears necessary to treat severely contaminated or macerated wounds after multiple debridement and irrigation procedures [9, 21].

### Pharmaceuticals

The most common effective intervention other than surgical management after trauma is the use of pharmaceuticals. Antibiotics are now generally recommended for wound and nosocomial infection prevention. In addition, tetanus vaccination, chlorhexidine, hydrocortisone, detrusor botulinum toxin A (BoNTA) injection, immunoglobulin, IFN-γ, and glucan have been noted in several studies and play an active role in preventing trauma-related infection.

#### Prophylactic antibiotics

According to the International Committee of the Red Cross (ICRC) Antibiotic protocol, the proper use of antibiotics is based on different types of injury [3]. Additionally, the US guidelines for the use of antibiotics in combat-related injuries suggest that post-traumatic antimicrobial agent selection and duration should be based on different combat-related injury patterns. For instance, antibiotic treatment for the following should be considered: extremity wounds (cefazolin, 2 g IV q6-8 h, 1–3 days), thoracic wounds (cefazolin, 2 g IV q6-8 h, 1 day after washout; if penetrating chest injury with esophageal disruption, metronidazole 500 mg IV q8-12 h is added), abdominal wounds (cefazolin 2 g IV q6-8 h with metronidazole 500 mg IV q8-12 h, 1 day after washout), maxillofacial and neck wounds (cefazolin, 2 g IV q6-8 h, 1 day), central nervous system wounds (cefazolin, 2 g IV q6-8 h, 5 days or until CSF; if contamination and abdominal cavity are involved, metronidazole 500 mg IV q8-12 h is added), and penetrating eye wounds (levofloxacin, 500 mg IV/PO once a day, 7 days) [8].

Many exploratory studies are drawing wide concern for the use of prophylactic antibiotics after trauma. Here, we list the basic principles.

- The time of administration Because the amount of bacteria increases exponentially from the time of trauma, 6 h appears to be a vital period after wound contamination. It is necessary to extend the time of antibiotic treatment if an unavoidable delay exists in administering antibiotics when the wound is open [4]. The ICRC suggests that penicillin, if it is not administered within 6 h in pre-hospital uncomplicated soft-tissue Grade 1 wounds, actually increases the risk of infection, which may be unavoidable [3].
- The choice of antibiotics Immediate treatment with broad-spectrum intravenous antibiotics based on the Gustilo and Anderson classification should be given in patients with open fractures or extensive soft tissue loss [11, 24]. High-dose intravenous 3rd-generation cephalosporins, rather than oral 1st-generation drugs, may be effective in patients with open fractures [25]. The incidence of wound infection in patients with fresh traumatic wounds or lacerations is low after the administration of co-amoxiclav [22, 26]. Cefazolin, or vancomycin if the patient is allergic to penicillin, cefoxitin/clindamycin and gentamicin, or clindamycin and gentamicin are commonly administered to patients in the trauma intensive care unit (TICU) [27].
- The course/dose of antibiotics Both civilian and military studies suggest that a short course and single dose of cephalosporins are important to prevent wound infection in open fractures and should be administered for either 3 days after injury or 24 h after wound closure [28]. Patients with penetrating abdominal trauma and concomitant thoracolumbar or sacral (TLS) fracture receiving prophylactic antibiotics for ≤ 48 hours do not develop spinal infections [29]. Prophylactic antibiotic research is applicable not only at the site of trauma wound infection but also in patients with nosocomial infections (early VAP and *Clostridium difficile* infection) [30, 31].
- The route of antibiotic administration There are several routes of antibiotics administration, depending on the different types of trauma. The administration of oral antibiotics is often applied to prevent wound infection for simple traumatic wounds [32, 33]. In addition, antibiotic ointments containing bacitracin, polymyxin, neomycin or cetrimide are often used in minor uncomplicated soft tissue wounds and have lower skin infection rates [34].

Overall, the recent studies concentrated on the prophylactic antibiotics after trauma are mostly retrospective and include the integration of expert opinions, but significant, random, double-blind, prospective studies are lacking.

#### Tetanus vaccination

The incubation period of tetanus is 3 to 21 days, and the risk of developing tetanus is great in any penetrating wound infection, especially in deep, small, punctate wounds. It is crucial for all trauma patients with deep wounds to receive appropriate immunization against tetanus. The present tetanus vaccination requirement, as a booster or revaccination, should also be considered by clinicians according to local protocols [22]. Because emergency clinicians are frequently faced with patients who are sensitive to tetanus infection in the emergency department, the attitude toward tetanus prophylaxis should be changed among emergency physicians [35].

#### Chlorhexidine (CHX)

For incontinence care, the involved skin should be wiped with as many chlorhexidine cloths as necessary after routine cleaning with soap and water. Chlorhexidine has been shown to be useful in reducing *Acinetobacter* colonization of the skin of ICU patients [8]. However, the effect of chlorhexidine on preventing nosocomial infection in trauma patients is controversial. Critically injured patients receiving daily bathing with 2 % chlorhexidine exhibited lower rates of catheter-related bloodstream infection and methicillin-resistant Staphylococcus aureus (MRSA) VAP [36]. Receiving chlorhexidine both from the time of admission to 48 and 72 h is also effective [37]. However, the administration of oral chlorhexidine over the first 48 h could not minimize the risk of VAP for intubated trauma patients [38].

#### Hydrocortisone

Adrenal insufficiency that alters organism immunity often occurs in severe trauma patients. Administration of an intravenous stress-dose of hydrocortisone has been associated with a lower incidence of hospital-acquired pneumonia (HAP) in ventilated patients with trauma [39, 40]. Subsequently, researchers have described the mechanism of hydrocortisone in a post-traumatic pneumonia mouse model; hydrocortisone can decrease trauma-induced immunosuppression by modulating the communication between DC and NK cells [41].

#### Other interventions

The most common clinical complication in patients with an indwelling catheter after spinal cord injury (SCI) is UTI. Neurogenic detrusor overactivity (NDO) is frequently detected in SCI patients and increases the risk of UTI. BoNTA injection may significantly reduce UTI in SCI patients with NDO and appears to decrease detrusor pressure [42]. In addition, immunomodulatory interventions such as immunoglobulin, IFN-γ, or glucan are the most effective in improving infection and MOF in trauma patients [43].

### Enteral nutrition (EN)

Receiving EN within 24 hours of severe injury and/or ICU admission can significantly reduce the pneumonia rate [44]. Some studies have indicated that both the nutritional quality and type of EN are critical for reducing hospital-acquired infections following trauma. High-quality EN formulas that contain omega-3 fats, extra levels of vitamins, minerals, and amino acids (such as glutamine) have been shown to reduce the rates of nosocomial pneumonia, bacteremia, abdominal abscesses, and UTI compared to standard EN [45]. The use of EN containing fish oil but not arginine is associated with a lower risk of secondary nosocomial infection [46]. EN with added probiotics is associated with a lower incidence of VAP [47]. In addition, transpyloric feeding (TPF) is associated with lower rates of VAP in severe TBI patients compared to gastric feeding (GF) [48].

### Management of the tube system

Practitioners placing central venous catheters in severe trauma patients should strictly observe sterile techniques to reduce the incidence of central line-associated blood stream infections (CLBSIs) [49]. Choosing the proper tube type is as critical as the indwelling time on intubation in trauma patients. Traumatic hemothorax with central venous catheter (CVC) placement has been associated with a lower infection rate of surgical wounds than a conventional large-bore chest tube [50]. Reducing the time of the indwelling urinary catheter could reduce the rate of UTI [51, 52].

### Organ dysfunction (OD) prevention

OD prevention has received increasing attention in patients with serious infections. Treatment with the calcium channel sensitizer levosimendan has been shown to be potentially advantageous for organ function in severe sepsis, especially on myocardial function [53–57]. Cardiopulmonary bypass (CPB), which is an essential cardiac surgery technique, appears to alleviate inflammation and prevent OD [58]. Critically ill patients who have an increased risk of extensive endothelial damage receiving autologous transplantation of endothelial progenitor cells (EPCs) may experience a restoration in blood flow, which could improve the function of important organs and thereby prevent MODS [59]. In addition, the Chinese medicine therapy of clearing-heat and detoxifying has also demonstrated beneficial effects on MODS prevention [60]. The effect of remote ischemic preconditioning (RIPC), which is a strategy to reduce ischemia in remote organs, has been controversial in renal injury [61]. Although the above pharmaceuticals and medical strategies have shown potentially preventive effects on OD, these measures have not yet been evaluated for use on trauma patients.

Some studies have reported several major risk factors in trauma patients leading to MODS, e.g., older age, the presence of chronic diseases, hypo-perfusion, infection, and immuno-depression [62–65]. To date, few effective interventions have been applied in OD prevention following trauma.

### Pharmaceuticals

Immunoglobulin, IFN-γ, or glucan may be effective in improving MOF in trauma patients [45]. Obese trauma patients (BMI >30 kg/m^2^) with an increased risk of MOF who received pre-injury angiotensin-converting enzyme inhibitor/angiotensin (ACE/ARB) therapy have markedly higher Marshall and Denver-2 scores compared to patients who did not receive these medications [66, 67]. Patients receiving either 7.5 % hypertonic saline (HS) or 7.5 % HS with 6 % dextran-70 (HSD) have a lower incidence of MODS than do patients receiving 0.9 % normal saline (NS) [68].

### Healthcare strategy

Patients with vascular injuries (arterial/ venous) at a Civilian Level I Trauma Center undergoing temporary intravascular shunt (TIVS) placement have lower rates of MOF and sepsis [69]. MOF appears to be prevented in critical multiple trauma patients on mechanical ventilation by using lung-protective strategies, avoiding high volumes and inspiratory pressure and improving the proportion of aerated lung during expiration. It has been shown that the rates of single organ failure, two organ failure and MOF are low [70]. The administration of a high-quality EN is important in trauma patients to prevent a nosocomial infection such as VAP or UTI [44–48]. The immunonutrition of EN and immune-enhancing diet also have demonstrated a vital role in reducing MOF following severe trauma. A combination of arginine, n-3-fatty acids and nucleotides has been associated with lower septic complications and lower MOF scores [71]. In addition, Chinese traditional treatment has potential validity in the prevention of OD. Acupuncture, which uses special thin needles that are pushed into the skin at particular points on the body, can activate vagal activity and cholinergic anti-inflammatory pathways and thereby improve the outcome of multiple traumatic patients. Liang et al. [72] reported that acupuncture at the ST-36 and PC-6 acupoints was associated with a lower incidence of systemic inflammatory response syndrome (SIRS), ARDS, sepsis and MOF.

## Conclusions

The initial intervention for sepsis following trauma is still a challenge. Early prevention for trauma patients can improve outcome and decrease mortality. Although many research studies about infection prevention in trauma patients have been published, there is a lack of guidelines for antibiotics after trauma, and some of the results are controversial. Currently, few effective interventions are applicable in the prevention of OD following trauma. Trauma can affect immunologic function, and wound infection, nosocomial infection, and secondary OD are all risk factors that are associated with sepsis following trauma. A new combination of measures can be generated to improve post-traumatic outcome. Overall, more efficient ways to prevent trauma-related infection and sepsis should be developed.

## Abbreviations

ACE/ARB: Angiotensin-converting enzyme inhibitor/angiotensin receptor blocker
ARDS: Acute respiratory distress syndrome
BoNTA: Botulinum toxin A
CHX: Chlorhexidine
CLBSIs: Central line-associated blood stream infections
CMA: Chinese Medical Association
CPB: Cardiopulmonary Bypass
CVC: Central venous catheter
DPC: Delayed primary closure
EN: Enteral nutrition
EPCs: Endothelial progenitor cells
GF: Gastric feeding
HAP: Hospital-acquired pneumonia
HS: Hypertonic saline
HSD: Hypertonic saline dextran
ICRC: International Committee of the Red Cross
MODS: Multiple organ dysfunction syndrome
MOF: Multiple organ failure
MRSA: Methicillin-resistant Staphylococcus aureus
NDO: Neurogenic detrusor overactivity
NPWT: Negative-pressure wound therapy
NS: Normal saline
OD: Organ dysfunction
RIPC: Remote ischemic preconditioning
SCI: Spinal cord injury
SIRS: Systemic inflammatory response syndrome
SOFA: Sequential Organ Failure Assessment
TICU: Trauma intensive care unit
TIVS: Temporary intravascular shunts
TLS: Thoracolumbar or sacral
TPF: Transpyloric feeding
UTI: Urinary tract infection
VAP: Ventilator associated pneumonia
WTD: Wet-to-dry
